# Indigenous Peoples’ Experience and Understanding of Menstrual and Gynecological Health in Australia, Canada and New Zealand: A Scoping Review

**DOI:** 10.3390/ijerph20136321

**Published:** 2023-07-07

**Authors:** Donna Ciccia, Aunty Kerrie Doyle, Cecilia H. M. Ng, Mike Armour

**Affiliations:** 1 NICM Health Research Institute, Western Sydney University, Sydney 2145, Australia; 2 School of Medicine, Western Sydney University, Campbelltown 2560, Australia; 3 School of Clinical Medicine, Health and Medicine, Division of Obstetrics and Gynaecology, University of New South Wales, Sydney 2052, Australia; 4 Gynaecological Research and Clinical Evaluation (GRACE) Unit, Royal Hospital for Women and University of New South Wales, Randwick 2031, Australia; 5 Jean Hailes for Women’s Health, Melbourne 3002, Australia; 6 Global Women’s Health, The George Institute for Global Health, Sydney 2042, Australia; 7 Medical Research Institute of New Zealand (MRINZ), Wellington 6021, New Zealand; 8 Translational Health Research Institute (THRI), Western Sydney University, Sydney 2145, Australia

**Keywords:** Aboriginal, Australia, Canada, dysmenorrhea, endometriosis, First Nations, Indigenous, menstruation, menarche, menstrual health literacy, New Zealand, scoping review

## Abstract

There are a variety of cultural and religious beliefs and customs worldwide related to menstruation, and these often frame discussing periods and any gynecological issues as taboo. While there has been previous research on the impact of these beliefs on menstrual health literacy, this has almost entirely been confined to low- and middle-income countries, with very little information on high-income countries. This project used the Joanna Briggs Institute (JBI) scoping review methodology to systematically map the extent and range of evidence of health literacy of menstruation and gynecological disorders in Indigenous people in the colonized, higher-income countries of Australia, Canada, and New Zealand. PubMed, CINHAL, PsycInfo databases, and the grey literature were searched in March 2022. Five studies from Australia and New Zealand met the inclusion criteria. Only one of the five included studies focused exclusively on menstrual health literacy among the Indigenous population. Despite considerable research on menstrual health globally, studies focusing on understanding the menstrual health practices of the Indigenous populations of Australia, New Zealand, and Canada are severely lacking, and there is little to no information on how Indigenous beliefs of colonized people may differ from the broader society in which they live.

## 1. Introduction

Women and those assigned female at birth are estimated to be nearly half of the world’s eight billion people. Of this, approximately 1.94 billion are of reproductive age (15–49 years) [1]. Menstrual health and gynecological disorders are common across the reproductive lifespan but especially prevalent among young adolescents occurring in 70–91% of teenagers [2,3,4,5], and can include primary and secondary dysmenorrhea (period pain), emotional changes, premenstrual syndrome (PMS), heavy menstrual bleeding, polycystic ovarian syndrome (PCOS), endometriosis, fibroids, or vulvodynia [6,7,8]. These menstrual health and gynecological disorders can impact all aspects of a person’s life, increase absenteeism and presenteeism (where people come in unwell and work less productively than normal) from work or school, and negatively impact a person’s quality of life, social engagement, relationships, and sports [7,8,9,10].

Menstrual taboos are still seen in many geographical locations, religions, and cultures worldwide [11,12,13], and despite approximately a quarter of the world’s population experiencing menstruation [14], this topic is often cloaked in silence [15,16]. Silencing contributes to poor health literacy around menstruation [14] and may disadvantage those affected. People with poorly managed menstrual health have a more significant impact on their lives compared to those with a better understanding of how to manage their symptoms [14]. The challenges and impacts of menstrual health on individuals and society are starting to be noticed and calls for action are occurring amongst the general public [17]. An example of this is grocery stores helping to debunk the stigma by renaming the shopping aisle “period care” instead of personal care or sanitary items [18] and organizations offering menstrual leave for their employees, a topic that is gaining traction internationally [19,20].

Poor menstrual health literacy can impact confidence, daily activity participation, education, and quality of life [21]. Indications of poor menstrual health literacy may include a lack of knowledge around the length of a menstrual cycle [22], what is considered “normal” when it comes to period pain and other symptoms [23], and the quantity of menstrual fluid lost each period [14]. Limited knowledge of over-the-counter pain medication dosing and the timing of taking these medications can often limit their effectiveness in controlling pain [24]. Knowledge of contraceptives, available period products and how to use and store them also pose a barrier to those with low menstrual health literacy [25]. We know that a lack of knowledge of a normal menstrual cycle leads to a delay in seeking help and achieving a timely diagnosis and implementation of a management or treatment program [21].

Understanding menstrual health (including the menstrual cycle) and its impact on a person’s social and physical life and psychological wellbeing [26] is essential for adolescents and their support network as they progress through the various stages of their reproductive life. The onset of menarche (onset of first menstruation) can be an incredibly challenging time for many adolescents. There may be a significant potential impact on their education [5] and overall quality of life during puberty, and can have ongoing effects on various aspects such as self-esteem, reproductive health, and life prospects [27].

To date, much of the menstrual health and literacy studies have been conducted in low-and-middle-income countries (LMICs), with less attention paid to higher-income countries (HICs) such as Australia, Canada, and New Zealand [21]. In HICs, young people and their parents often have similar difficulties in comfortably discussing menstruation [7,28], leading to poor menstrual health literacy [14]. Given that there can be significantly different levels of health literacy and health equity amongst different cultural and religious groups, it is possible that health literacy may be lower in some of these groups due to systemic and other barriers [27].

It is important to note that the Indigenous groups included in this study have different religious and cultural beliefs and should not be considered a homogenous group; however, health inequity, life expectancy, and socioeconomic disadvantages are common amongst these groups [29,30,31].

Indigenous people in Australia are of Aboriginal and Torres Strait Islander heritage who are the First Nations Peoples of Australia, with hundreds of First Nations language groups being included in this overarching group. Previous research has identified that there is a gap in health equity amongst the Australian Aboriginal and Torres Strait Islander people [32,33]. According to the Australian Government’s Closing the Gap Report [34], the life expectancy for Indigenous Australians is not improving at the forecasted rate, and in fact, there is further widening of this health gap for First Nations people. Currently, Indigenous Australians are offered an annual Indigenous Health Check and follow-ups, but the uptake in this program is reported to be 28% of the Indigenous population [35]. The purpose of the annual health checks is to manage chronic disease conditions; with other health concerns expected to be initiated by patients [36]. The delay in healthcare poses many issues for the Indigenous community, and the rates of many diseases are higher in Indigenous Australians than those from a non-Indigenous background. Chronic conditions such as cardiovascular disease, diabetes, and kidney disease are significantly more likely to occur in Australian First Nations people than in the non-Indigenous population [37].

Canadian Indigenous peoples are divided into three distinct groups, the First Nations, Inuit, and Métis, and collectively account for more than 1.67 million people in the 2016 Canadian Census. Like the Australian Indigenous population, the Indigenous people in Canada also have a substantial socioeconomic disadvantage compared to their non-Indigenous counterparts [38] in both a historical and contemporary context. Examples of health disparities in Indigenous people are highlighted by the higher rates of type 2 diabetes, hypertension, post-surgery mortality and complications, and overall lower health outcomes [39].

Similarly, the Aotearoa New Zealand’s Māori Indigenous population, which accounts for around 17% of the total population [40], have persisting health inequalities, including higher rates of heart disease and diabetes [41], and has limited engagement with health services [42,43].

It should be acknowledged that not all people who menstruate experience painful menstruation, and not all period pain is primary dysmenorrhea. Secondary dysmenorrhea is common, and one of these causes may be attributed to a disease such as endometriosis. The normalization of pain during menstruation has often led to delays in seeking help and timely diagnosis [44,45]. Crucially, given the long-standing health inequalities, this delay may be more significant in Indigenous people [46]. Recognizing ‘abnormal’ menstrual symptoms (such as dysmenorrhea, heavy menstrual bleeding) and diagnosis of associated gynecological health conditions or pathologies, such as endometriosis, needs to be balanced with improved pathways to access educational information and expert care [47]. A priority under the Australian Government’s National Action Plan for Endometriosis [48] is the identification and support to develop endometriosis-specific education and awareness materials that are tailored for Aboriginal and Torres Strait Islander communities.

We currently have limited information about how menstrual health, menstrual health literacy, and gynecological disorders may affect Indigenous peoples; this scoping review aims to systematically map the extent and range of current evidence of menstrual health literacy in this cohort in Australia, Canada and New Zealand. This scoping review will help identify the research gaps and lay the foundation for potential future research in this area. Future co-designed research can have practical implications for communities worldwide, including access to culturally safe education, healthcare, and treatment options.

## 2. Materials and Methods

### 2.1. Research Question

The research question we aimed to answer with this scoping review was, “What evidence is available on the experience and understanding of menstrual health in the Indigenous peoples of Australia, Canada, and New Zealand?”.

### 2.2. Study Design

Our research question and objective were best suited to a scoping review, given that we aimed to examine the research that was available on understanding menstrual health literacy amongst Indigenous peoples of Australia, Canada, and New Zealand. These countries were chosen as they were (a) colonized, (b) high-income countries, (c) have English as an official or principal language, and (d) have similar publicly funded healthcare systems. It is expected that the results arising from this scoping review will provide and assist in mapping the extent and types of research studies already existing and identify the knowledge gaps by utilizing the methodology as described by Levac et al. [49].

**Figure 1:**
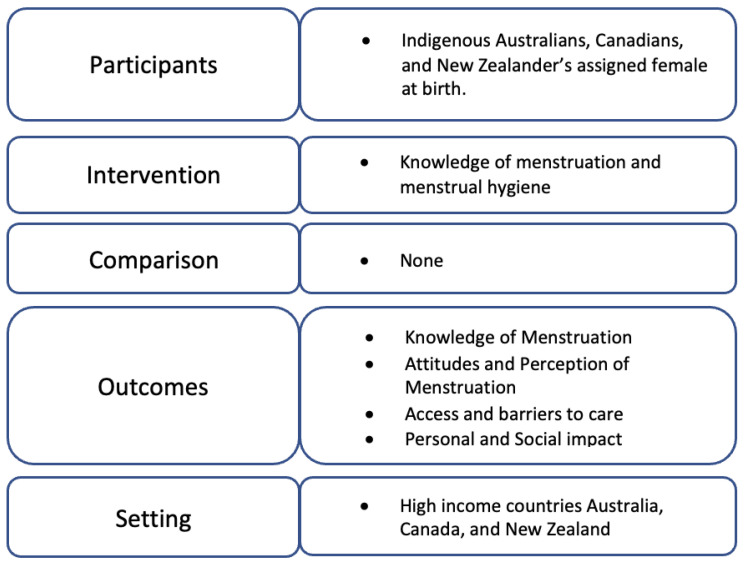
Populations, interventions, comparators, outcomes, and setting (PICOS) criteria for inclusion.

The Joanna Briggs Institute (JBI) scoping review [50] methodology was followed, which contains nine steps and expands on the methodological framework of Arksey and O’Malley [51] and Levac et al. [49] for conducting a scoping review. The review focuses on the evidence relating to Indigenous Peoples’ experience and understanding of menstrual health (Figure 1).

### 2.3. Search Strategy

The search strategy aimed to identify both published and grey literature. An initial search of PubMed, PsycInfo, and CINAHL was undertaken to identify articles on the topic in March 2022. The words contained in the titles and abstracts of relevant articles and the index terms used to describe the articles were used to develop a full search strategy for PubMed, CINAHL, and PsycInfo. The search strategy included all identified keywords and index terms; it was adapted for each included database and/or information source. The keywords used were kept broad in scope to help identify a pool of studies, as we anticipated there would be a limited quantity of research in this area. The reference list of all included sources of evidence was screened for additional studies. A grey literature search was also conducted.

We systematically searched three electronic databases (PubMed, CINAHL, and PsycInfo) and three grey literature electronic databases—Trove (Australia) Newspapers and Gazettes; Magazines and Newsletter; Images, Maps, and Artefacts; Research and Reports; Books and Libraries; Diaries, Letters, and Archives; Music, Audio, and Video; People and Organizations; Websites, EBSCO Open Dissertations and ProQuest Dissertations and Theses A&I using the search terms in Table 1.

Only studies published in the English language were included, as this is the common and/or official language for the selected countries. All studies were included irrespective of date.

### 2.4. Study Selection

Following the search, all identified citations were collated and uploaded into EndNote 20 (Clarivate Analytics, Philadelphia, PA, USA), and duplicates were removed. Following a pilot test, titles and abstracts were then screened and assessed against the inclusion criteria for the review.

**Figure 2:**
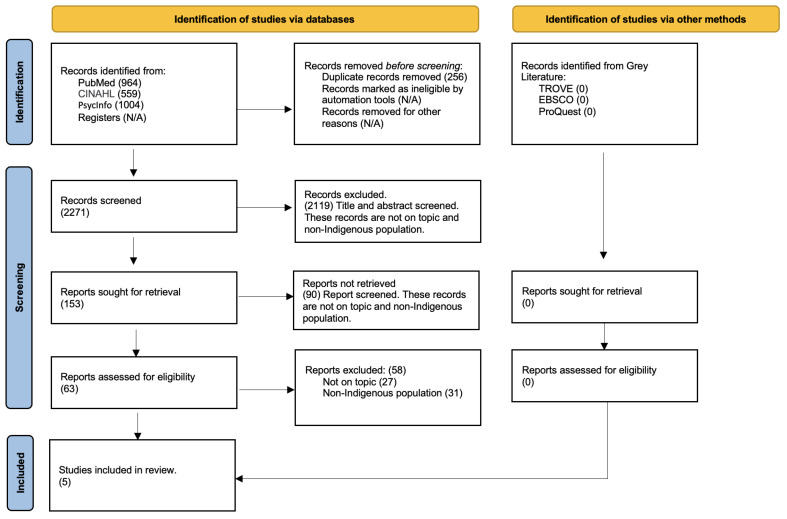
PRISMA flow diagram of search screening results for a scoping review.

The full text of selected citations was assessed in detail against the inclusion criteria. Reasons for the exclusion of sources of evidence in full text that did not meet the inclusion criteria were recorded. The results of the search process and the studies included in this scoping review are reported in full and presented in a Preferred Reporting Items for Systematic Reviews and Meta-analyses extension for a scoping review (PRISMA-ScR) flow diagram [52] (Figure 2).

For inclusion, studies must have the following:

- Included Australian, Canadian, and New Zealand Indigenous people (women and those assigned female at birth).
- The main subject area is related to menarche or menstruation or menstrual health disorders (including dysmenorrhea, premenstrual dysphoric disorder (PMDD), etc.) or gynecological disorders (such as PCOS, adenomyosis, endometriosis, etc.).
- Measured at least one of these outcomes and be connected to menstrual experiences, defined as:
  ○ Personal knowledge, thoughts, feelings, beliefs, narratives;
  ○ Practices related to menstruation and menstrual health disorders;
  ○ Impact of menstruation on daily living;
  ○ Pathways to treatment opportunities.

Studies were excluded if they focused on the subject areas of general puberty, not explicitly relating to menstruation and issues relating to period poverty and access to menstrual care products. Studies were also excluded if they addressed menstruation but did not address menstruation as the primary area of study (e.g., amenorrhea, developmental or behavioral co-morbidities and their impact on menstrual hygiene and health). Studies on malignant gynecological conditions such as cervical cancer were also excluded.

### 2.5. Data Charting and Synthesis

Extracted data relevant to our research question, included specific details from the included studies: study population characteristics, concept, context, study methods and key findings under four topic areas ((i.) knowledge, (ii.) attitudes/perceptions, (iii.) access and barriers, and (iv.) personal and social impact of menstrual health literacy).

## 3. Results

Our research returned 2271 studies, and we identified five studies from two countries, Australia and New Zealand which met all the inclusion criteria. One Australian study exclusively included Indigenous people [27], and four studies, three from Australia [10,14,53] and one from New Zealand [36], included a mix of Indigenous and non-Indigenous peoples. No studies from Canada met our inclusion criteria.

The results were organized using our inclusion criteria topic areas: (i) knowledge of menstruation, (ii) attitudes and perceptions of menstruation, (iii) access and barriers, and (iv) personal and social impact. The characteristics of the included studies (Table 2) are a mix of quantitative and mixed methods using both community settings and online recruitment. Table 3 provides the data extracted and information from each of the five included studies in this scoping review; the raw data are provided in Supplementary Materials, Table S1.

### 3.1. Knowledge of Menstruation

Overall, the five studies all highlighted that the lack of knowledge of menstruation and menstrual health hygiene and literacy was common. Studies that included both Indigenous and non-Indigenous people showed little to no difference in levels of menstrual health literacy between these two groups, while those that looked at Indigenous people only noted how difficult it was to untangle the influence of post colonization social and medical beliefs from traditional beliefs. Krusz and colleagues (2019) [27] called attention to the limited availability of current studies on menstrual knowledge in the Indigenous communities, including hormonal contraception and menstrual disorders. Armour and colleagues (2021) [14] highlighted an interesting point that despite the Australian curriculum, including reproductive health, there is limited knowledge on this topic, highlighting the potential ineffectiveness of the current program [14]. In the many instances where there was a significant limited knowledge of menstruation and menstrual health literacy, this often led to the delay in diagnosing chronic health conditions such as endometriosis or other pathologies causing secondary dysmenorrhea [36] in both Indigenous and non-Indigenous people. These diagnostic delays in chronic health conditions can negatively impact social life, academic and work performance, fertility, and relationships [14].

The four concepts identified in the area of knowledge are ‘private women’s business’, ‘under-resourcing for education’, ‘sources of knowledge’, and ‘diagnostic delay’.

The study by Krusz and colleagues (2019) [27] highlighted that menstruation is considered a ‘private women’s business’ in many First Nations communities, making it an especially sensitive discussion topic. Lansbury and colleagues (2021) [53] identified the lack of knowledge of puberty-related changes, product preferences, and hormonal contraceptives and cultural shame was associated with menstruation.

Krusz and colleagues (2019) [27] reported a demand for every health education resource available, particularly for chronic diseases such as diabetes, to be made culturally relevant for Indigenous communities. As all health conditions are under-resourced for educational materials and because menstruation is not seen as an illness or disease, it was therefore categorized as a low priority in health education, particularly for remote locations. Not having access to menstrual health education was found to be a severe concern impacting girls’ and women’s lives, including being unable to go to school [27]. A study by Armour et al. (2020) [10] of non-Indigenous and Indigenous participants, reflecting the national Indigenous population of 3.3%, identified that 49.8% of the participants indicated that they used online information to determine whether their period was normal. This lack of understanding of what ‘normal’ and ‘abnormal’ menstruation is has highlighted the need for more education and access to information, including culturally appropriate information.

Sources of knowledge on menstruation were identified by Armour and colleagues (2020) [10]. Discussing the topic of menstruation identified that the participants’ mother was seen by over a third (36.3%) of the participants to be their source of menstrual information. This number was slightly less (31.1%) for those speaking with their doctor, despite the recognition by participants that health professionals are the most trustworthy source of information. Notwithstanding the array of online and in-person menstrual education programs, this group of adolescents and young adults overall had limited ability to manage period pain and menstrual health literacy [10].

In a second paper from Armour and colleagues (2021) [14], the authors outlined the sources of knowledge for adolescents and young adults. According to the finding, approximately 49.8% of the respondents sought online sources to determine whether their period was normal. Thirty-six percent of the 4202 respondents discussed this topic with their mothers, and only 31% spoke with their doctors for their menstrual health information. However, health professionals were considered the most trustworthy source of information, followed by family and the internet, with only 20.7% of participants thinking their teachers were a trusted source of information [14].

In Tewhaiti-Smith and colleagues (2022) [36], a multi-ethnicity study into endometriosis and CPP identified that between the onset of the first symptoms and their presentation to a doctor, there was a delay between 2.4 to 4 years. Furthermore, despite multiple visits to their doctor, it was identified that there is an average of eight years of diagnostic delay for endometriosis. Currently, menstrual (or reproductive) health education is driven by advocacy groups running individual programs, as there is no formal compulsory educational curriculum focusing on CPP pain in Aotearoa, New Zealand [36].

### 3.2. Attitudes and Perception of Menstruation

Feelings of shame and a sense of secrecy were common attitudes and perceptions associated with menstruation and were described by all five studies. It was also illustrated that menstruation had a detrimental impact on education, with impacts from presenteeism through to absenteeism at school. These perceptions also impacted on attitudes towards medical information-seeking behavior and contributed to delays in diagnosing gynecological conditions. Furthermore, despite current teachings, the onset of menstruation triggered negative social stigmas, impacting self-esteem.

The central concepts of ‘shame’, ‘secrecy’ and ‘private women’s business’ were consistently reported to be associated with menstruation across all five studies in this scoping review [10,14,27,36,53]. This stigmatization was identified as being a potential factor preventing women and girls from living shame-free every month during menstruation [27]. Both Western and Indigenous cultural influences were also described as potentially influencing the stigmatization and inability to openly discuss menstruation and bleeding openly [27,53]. It was highlighted by Lansbury and colleagues (2021) [53] that Indigenous women often easily feel shame when discussing menstruation.

Three studies described the attitudes and behaviours when seeking medical advice relating to menstruation [10,14,36]. Armour and colleagues (2020 and 2021) [10,14] identified that the internet was a significant source (50% of respondents) of information when seeking advice on menstrual symptoms. With 51% of participants of Armour and colleagues (2020) [10] research participants describing their period as “normal” despite experiencing significant dysmenorrhea. It was also reported that self-management strategies with over-the-counter analgesia medications and oral contraceptives were commonly engaged practices to reduce pain, 51% and 35%, respectively [14]. Tewhaiti-Smith and colleagues (2022) [36] state that such attitudes and health-seeking behavior may exacerbate the delay in diagnosing gynecological conditions in the Māori population.

### 3.3. Access and Barriers

Barriers were reported across all five studies, including internalized taboos, cultural secrecy and shame about the natural bodily processes and physical barriers such as shared accommodation and access and disposal of menstrual products. Limited menstrual knowledge has a far and lasting impact on individuals and communities, such as negative education impacts, including absenteeism and reduced quality of work, as highlighted by Armour and colleagues (2020) [10].

Lansbury and colleagues’ (2021) [53] study identified 16 barriers divided into four clusters and further divided these barriers into age groups to highlight the priority barriers for each age group. The first barrier the authors identified was structural, highlighting living situations consisting of busy households, access to products, waste disposal, multiple homes, bodily hygiene, and disabilities [53]. Krusz and colleagues (2019) [27] also identified structural barriers: storing and transporting products, overcrowded housing, multiple residences and the lack of privacy to keep personal items safely.

The second cluster identified by Lansbury and colleagues (2021) [53] was knowledge, culture, and behaviour: knowledge, shame, puberty-related changes, product preferences, and hormonal contraceptives [53]. Krusz and colleagues (2019) [27] also noted that buying menstrual products can be embarrassing and expensive—‘People are not going to the shop and buying it because they are tiny places and people will know you have bought it because you are menstruating… there is sort of a bit of …stigmatizing or feeling ashamed’ (Research representative #2) [27]. Knowledge was also identified by Armour and colleagues (2020) [10], who discusses that despite access to menstrual education through the Australian curriculum, the effectiveness of these education programs is yet to be assessed. The research by Armour and colleagues (2021) [14] highlighted that the lack of basic knowledge of menstruation and low health literacy led to associated overall poorer health outcomes.

Lansbury and colleagues (2021) [53] described the third cluster as discomfort and public life: pain and hormonal moodiness, and school attendance in discomfort. Other practical concerns, such as having enough sanitary products during their day at school or university, bleeding through their clothes and how to conceal the products, were reported by Armour and colleagues (2020) [10]. Furthermore, the research by Armour and colleagues (2021) [14] discusses that the timing of medication could be a barrier to effective pain management. Over-the-counter medications were often taken after the pain had started, and those that took mefenamic acid were likely taken before the onset of pain.

The fourth and final cluster identified by Lansbury and colleagues (2021) [53] was finances which highlighted the cost of menstrual products and household purchase priorities. The research by Tewhaiti-Smith and colleagues (2022) [36] identified that in Aotearoa New Zealand, there were numerous barriers to accessing healthcare which may have an impact on individuals’ ability to navigate the health system to diagnose and manage their CPP or endometriosis. These barriers included practitioner bias, logistics, financial, and notable inequities, with ethnic minorities experiencing poorer health outcomes. Unmet patient needs are noticeable in the current healthcare model for CPP sufferers [36].

### 3.4. Personal and Social Impact

The potential impact, both personally and socially, is wide reaching. The lack of menstrual literacy can leave lasting effects and challenges for the individual, such as delays in diagnosis, absenteeism from education and employment, and a negative impact on relationships.

Krusz and colleagues (2019) [27] stated that menstruation is an under-acknowledged challenge that impacts the health and wellbeing of women and girls as well as their broader communities. The impact on girls’ self-esteem, sexual and reproductive health, and school attendance are believed to be linked to menstrual resource (un)availability and the sociocultural environments with a lasting effect on women’s health outcomes for the course of their lives.

Armour and colleagues (2020) [10] study identified a detrimental impact on education with absenteeism, presenteeism, and concentration problems. They identified that concentration in the classroom during menstruation was impacted for the better part of the school and tertiary education. Absenteeism rates were directly correlated to those with high pain scores and reduced performance in the classroom. Lansbury and colleagues (2021) [53] highlighted their yarning circles discovered that overall, girls from all the year groups described pain and mood issues as moderate to significant barriers associated with managing their periods. The impact on education was potentially significant. Most of the people whose academic performance was negatively impacted would not speak to teaching staff about their experience with menstruation [10].

Tewhaiti-Smith and colleagues (2022) [36] study also identified an impact on education, work and relationships, including sexual and personal. Their research shows there was an impact on education due to endometriosis and CPP symptoms, demonstrating there were days lost each month to studying and some giving up altogether. The majority of respondents reported an impact on their employment, with over 70% having either lost their jobs, having to change jobs or reduce their working hours due to the impact of symptoms. The study by Armour and colleagues (2021) [14], discovered that the self-management characteristics of participants led to ill-timing and sub-therapeutic doses of medications rather than seeking professional medical advice. This sub-optimal therapeutic dosing, in turn had a significant negative impact on academic engagement and extracurricular activities.

Krusz and colleagues (2019) [27] described that stigma surrounding menstruation also had a broader effect of marginalizing women’s health concerns from menstrual disorders to menopause [54,55]. Menstruation has an impact on girls’ self-esteem, sexual and reproductive health, and school attendance. Lansbury and colleagues (2021) [53] highlighted that the onset of menstruation in different cultures could trigger ceremonies, teachings, and celebrations with the entry into womanhood. However, these discussions can often trigger negative social stigmas that can have ongoing social esteem, body image, and hygiene-related health issues [56]. Lansbury and colleagues (2021) [53] also discussed that shame and secrecy are socially imposed experiences for many people, which can result in significant disruptions in their lives.

## 4. Discussion

Our review found a lack of research into, and therefore knowledge of, the experience and understanding of menstrual and gynecological health for Indigenous people in Australia, Canada, and New Zealand. Only five studies were found that included Indigenous people from Australia, Canada, and New Zealand, with only one of these focusing on Indigenous people specifically. No studies were identified for the Indigenous people in Canada. Due to this limited information, we have very little understanding of the lived experience and challenges of these Indigenous communities in dealing with menstruation and menstrual health literacy and how their challenges may differ from the society in which they live post colonization. Previous research shows that menstruation is viewed through a cultural lens [21], but in this instance, the included studies do not report on any customs or rituals, and any challenges these may bring and how these challenges may be managed or resolved. It falls substantially behind the pace of research for other groups around the world and the increased awareness of the importance of menstrual health knowledge [57]. This review indicates that Indigenous people from Australia, Canada, and New Zealand are being left behind, and the already significant health gap remains and may widen unless steps are taken to address this [32,33,34,38,43,58].

Much of the research on menstrual health literacy in LMICs has identified an overall fundamental lack of general knowledge about menstruation and what is considered “normal” [59]. Our review found that despite residing in HICs, where sexual health education is widely available in schools [10,60,61] and where discussion of menstruation is expected to be less taboo [21], there was little to no representation in the literature for Indigenous peoples and their experiences of menstrual and gynecological health. The paucity of research evidence is disproportionate to the scale of the issues associated with menstrual health, education, and gynecological disorders and their management [3]—and small in comparison to the volume of evidence for the non-Indigenous community [5,61]. This may be reflective of the lack of funding and engagement and the challenges that may surround research in these groups. There is no existing research either to understand or assess the quality, cultural safety, and efficacy of any education specific to any of the different Indigenous populations included in this review.

Health disadvantages are well documented in Indigenous communities [32,38,39,41]. Identified menstrual health disadvantages in LMICs and HICs [21] are often reflected broadly in the communities. Challenges arising from ‘secret women’s business’ such as accessing, purchasing, storing, and concealing menstrual products through to disposing of these products [53] highlights some of the issues related to menstrual health faced by Indigenous women and those assigned female at birth. The associated culture, behaviors, and shame around menstruation are compounded for some by living in busy households across multiple dwellings with multiple people and absenteeism from school. It was noted that the topic of menstruation is often not even discussed amongst the Indigenous community themselves [53].

The challenges experienced by many people are often exacerbated due to the silence this normal bodily process creates [26], which may be heightened in some cultures, such as in the Indigenous populations of Australia. Feelings of shame are often internalized, and secrecy often leads to lateness, incompleteness, or total lack of access to health information [27], and this barrier has implications for mental health and social, education, and workplace absenteeism [21]. An interesting finding was that despite the Australian school curriculum including reproductive health topics, there still remained limited knowledge on this topic, irrespective of whether students were Indigenous or not, which highlights the need for an evaluation of the effectiveness of the current program to ensure these programs are designed to tackle these culturally specific issues [14]. Assessment of the quality and efficacy of the menstrual health education programs delivered within the Australian curriculum and programs provided by private educators should be performed to determine the effectiveness and awareness of menstrual health and associated gynecological disorders, as there is evidence to suggest that these are not meeting the needs of young people [62].

There have been challenges with engaging these groups of people in research in the past, as a colonial lens has been commonly used to conduct research on Indigenous people instead of developing research partnerships being led by Indigenous people [63]. This has caused multi-generational harm with inter-generational trauma and mistrust of government, researchers, and health professionals [64]. Most of the included studies in this review either were led by Indigenous researchers, as in the case of Tewhaiti-Smith and colleagues (2022) [36] or using a participatory approach, including Indigenous facilitators and culturally appropriate techniques, such as yarning circles in the case of Krusz and colleagues (2019) [27]. Lansbury and King (2021) [53] used yarning circles where one of the authors, Minnie King, a key figure in the local Indigenous community where the research was undertaken, facilitated these. The other two studies did not outline their approach to engaging the Indigenous community, and this is likely due to the fact that these studies aimed to collect a broad national sample rather than focusing on Indigenous people specifically [10,14]. 

Culturally safe and appropriate menstrual health education, which respects customs and people, is essential for respectful community engagement [65]. The evidence around sociocultural norms surrounding menstruation and its function in a multi-cultural community is critical to inform the design and content appropriate for Indigenous groups. Therefore, educational programs that cover menstrual health need to ensure that all subsequent versions of these programs include input from Indigenous people and, where relevant, other culturally and linguistically diverse peoples, to ensure their needs are addressed in culturally appropriate and sensitive ways. Outlining a set of standards, key concepts, and goals to be achieved is essential to be culturally safe and respectful of customs and people.

According to the Australian Institute of Health and Welfare, Indigenous females in Australia tend to be younger than non-Indigenous females, with around 34% aged under 15, compared with around 18% non-Indigenous females (ABS 2018a) [66]. This larger Indigenous youth population seems to be mirrored in Canada [58] and New Zealand [40]. This younger population of Indigenous peoples gives rise to an opportunity for collaboration, innovation, and education for a new generation of Indigenous peoples to change the current status quo of health inequalities. These younger populations highlight the current opportunities and urgency to act now to discover the cultural experiences, knowledge of customs, and barriers to improving outcomes for these younger populations to change the trajectory of poorer health and life outcomes. 

The studies included in this scoping review provided only brief information on self-management practices undertaken. Lansbury and colleagues (2021) [53] reported the use of rest, heat, pain medication, and exercise amongst Indigenous Australians. This is similar to the self-management strategies used in Australia [14] and worldwide [67]. Given that there are Indigenous healing practices such as Rongoā Māori (traditional Māori medicine that includes herbal remedies, physical therapies, and spiritual healing) [68], it would be vital to understand how different Indigenous groups might utilize these traditional health practices as part of menstrual health and gynecological disorder management. Previous research has described that such culturally specific remedies are common in the self-management of menstrual symptoms [14]. This engagement would pave the way to empower a younger generation with knowledge of menstruation and potentially shorten the delay of diagnosis for gynecological conditions such as endometriosis, which is a leading cause of secondary dysmenorrhea [67,69].

There are several practical implications from these findings for future research. Research amongst Indigenous people should adhere to the principle of “Nothing about us, without us” [70], where there must be explicit input from the population that will be affected by the research outcomes. However, given that research on Indigenous or minority groups historically has often been ethically problematic, at best, it is unsurprising that many Indigenous groups are reluctant to participate in research [71]. In addition, the approach to consumer engagement in countries such as Australia has been noted by other researchers as being “piecemeal” [72]. One potential solution is ensuring engagement with local community elders or leaders; however, as others have noted, a partner approach to co-design is not necessarily ideal [73] and that Indigenous-led and Indigenous designed/developed [74] research is preferable, as this supports capacity building amongst Indigenous researchers [75]. Therefore, our key recommendation from this review is that Indigenous voices must be included at all stages of the research—from design to data interpretation and implementation, ideally as Indigenous-developed and led research. 

This scoping review has several limitations. Firstly, we searched for articles that discussed menstruation and specific gynecological conditions such as endometriosis; however, other studies that may have reported on these issues, where this was not the main focus of the study, could have been missed. The reference lists of the included studies were also reviewed to identify any potential studies that may have been missed in our search, and none were found. Secondly, all searches were undertaken in English only. English is the primary language (Australia) or one of the official languages (Canada and New Zealand), and so while unlikely, it is possible that studies in another language may have been missed. 

## 5. Conclusions

Very few relevant studies were found related to menstrual health literacy or gynecological health amongst the Indigenous people in Australia, New Zealand, and Canada. Only a single study specifically examined Indigenous experiences and beliefs, and overall studies demonstrated a similar level of menstrual health literacy as young people in general. Due to the paucity of research, it is difficult to determine what, if any, cultural beliefs and customs influence menstrual knowledge and symptoms management, if these differ from the wider society they live in, and if these beliefs have been influenced by colonization. This review shows that there is a need for more investment in Indigenous-led or, where this is not possible, appropriately co-designed research in this area to ensure that these communities and groups have access to accurate, culturally relevant information on menstruation. 

## Figures and Tables

**Table 1:** Search terms for individual databases.

Databases	Search Terms
CINAHL	(menarche OR menstrua* OR dysmenorrhea OR endometriosis) AND (Indigenous OR aboriginal OR first nations OR maori OR torres OR canada OR australia OR new Zealand)
PsycInfo	(menarche OR menstrua* OR dysmenorrhea OR endometriosis) AND (Indigenous OR aboriginal OR first nations OR maori OR torres OR canada OR australia OR new Zealand)
PubMed	(menarche OR menstrua* OR dysmenorrhea OR endometriosis) AND (Indigenous [Title/Abstract] OR aboriginal [Title/Abstract] OR first nations [Title/Abstract] OR maori [Title/Abstract] OR torres [Title/Abstract] OR canada [Title/Abstract] OR australia [Title/Abstract] OR new zealand [Title/Abstract])
Grey Literature	Menarche, Menstrual Cycle OR Menstruation Disturbances OR menstrua* OR Menstruation; Menstrual Cycle; Dysmenorrhea; Endometriosis; Aboriginal Australians; Torres Strait Islanders; First Nations of Australia; Oceanic Ancestry Group; Indigenous Australian; Australian Aborigine; Indigenous Australians; Aboriginal; Indigenous Peoples—similar relevant terms for Canada and New Zealand.

* truncation of root term in literature search.

**Table 2:** Characteristics of included studies.

Country	Australia (4)
	New Zealand (1)
	Canada (0)
Outcome measure	Knowledge of menstruation (5)
	Attitudes and perception of menstruation (5)
	Access and Barriers (5)
	Personal and social impacts (5)
Study type	Qualitative—Co-design (2)
	Quantitative—Cross sectional online survey (3)
Study population	Mature Aged—assigned female at birth (1)
	10–18-year-olds—assigned female at birth (1)
	13–25-year-olds—assigned female at birth (2)
	18–74-year-olds—assigned female at birth (1)

**Table 3:** Key findings from the studies (*n* = 5) included in this scoping review.

Study ID	Study Characteristics		Study Participants								
	Study Design	Methodology	Age Range	Race/ Ethnicity	Socio-Economic Status	Region	Population	1. Knowledge	2. Attitudes/Perception	3. Access and Barriers	4. Personal and Social Impact
Krusz (2019) [27]	Co-designed qualitative	Qualitative—Yarning circle	Mature age demographic	Australian First Nations People	Low socio-economic	Brisbane (Queensland, Australia)	*n* =12 Aboriginal and Torres Strait Islander women and *n* = 8 non-Indigenous co-researchers and practitioners from urban, rural and remote areas.	Lack of contemporary, comprehensive puberty education that includes information on menstruation and menstrual hygiene.	Menstruation is considered private ‘women’s business’ in many Aboriginal and Torres Strait Islander cultures, making it a particularly sensitive topic to discuss. Stigma, secrecy, and shame involved with discussing menstruation and bleeding may prevent older women from exploring those issues with young people.	Barriers: storing and transporting products, overcrowded housing, multiple residences, lack of privacy for safe keeping of personal items. Financial barriers.	Impact on girls’ self-esteem, sexual and reproductive health, and school attendance. Need to improve MHH without distraction from engaging in full societal participation.
Lansbury (2021) [53]	Co-designed qualitative	Qualitative—Yarning circle	10–18 years and adults supporting students.	Indigenous and non-Indigenous girls	Low socio-economic	Weipa (Far North Queensland, Australia)	Yarning circles with *n* = 72 plus *n* = 15 adult interviews; (*n* = 7 Indigenous people and *n* = 8 non-Indigenous people).	Lack of knowledge, puberty-related changes, product preferences, hormonal contraceptives, and cultural shame associated with menstruation.	Shame is often easily felt by Indigenous women when discussing menstruation. Stigma, secrecy, and shame are involved with discussing menstruation and bleeding.	Barriers to accessing and disposing of menstrual products.	Educational impact was potentially significant.
Armour (2020) [10]	Quantitative	Quantitative—Cross sectional online survey	13–25 years—(median range 17 years).	Australian Indigenous and Non-Indigenous	37% lived in low socioeconomic area and 16% lived in a high socioeconomic area.	Australia	*n* = 4202	Limited knowledge of menstruation.	Affected by both presenteeism and absenteeism with a detrimental impact on education. Menstrual stigma and a variety of ethical and cultural challenges.	Access to menstrual education is available but effectiveness has not been assessed.	Detrimental impact on education with both absenteeism and presenteeism, and concentration problems. Higher menstrual pain scores correlated to higher impact.
Armour (2021) [14]	Quantitative	Quantitative—Cross sectional online survey.	13–25 years—(median range 17 years).	Australian Indigenous and Non-Indigenous	37% lived in a low socioeconomic area and 16% lived in a high socioeconomic area.	Australia	*n* = 4202	Limited knowledge of menstruation and health literacy. Knowledge gained from the internet.	Participants reported problems with classroom concentration during menstruation: menstrual stigma and a variety of ethical and cultural challenges.	Lack of basic knowledge of menstruation and low health literacy leading to associated overall poorer health outcomes.	Impact is most women self-managing their symptoms with the use of ‘over the counter’ pain medications and hormonal contraceptives.
Tewhaiti-Smith (2022) [36]	Quantitative	Quantitative—Cross sectional online survey	18 and over (median 31.8 years)	European and Māori	Most reported university level education and most respondents were in the $501–$1500 (NZD) per week earnings.	Aotearoa New Zealand	*n* = 800	Limited and often delayed diagnosis.	Endometriosis and CPP a negative impact to work, daily activities, and quality of life. Limited awareness	CPP often delayed diagnosis and increased the economic burden.	Impact of diagnostic delay—significant effects on all social domains, education, work, and sexual and other personal relationships.

## Data Availability

Data is contained within the article or Supplementary Materials.

## Supplementary Materials

The following supporting information can be downloaded at: https://www.mdpi.com/article/10.3390/ijerph20136321/s1, Table S1. Raw extracted data from the included studies in this scoping review.
