# Supplementary material for: Indigenous Peoples’ Experience and Understanding of Menstrual and Gynecological Health in Australia, Canada and New Zealand: A Scoping Review
Source: Int J Environ Res Public Health. 2023 Jul 7;20(13):6321. doi: 10.3390/ijerph20136321 (PMC10341312; doi:10.3390/ijerph20136321)
Supplement: Supplementary file 1 [file ijerph-20-06321-s001.zip › ijerph-2130974-supplementary.pdf]

SUPPLEMENTARY MATERIALS

Table S1. Raw extracted data from the included studies in this scoping review.

	Reference Information								
	**PubMed ID**	**Title**	**Authors**	**Citation**	**First Author**	**Abstract**	**Keywords**	**Journal/ Book**	**Publication Year**
Krusz (2019)	PMCID: PMC6882156	Menstrual health and hygiene among Indigenous Australian girls and women: barriers and opportunities	E. Krusz, N. Hall, D. J. Barrington, S. Creamer, W. Anders, M. King, et al.	Krusz E, Hall N, Barrington DJ, et al. Menstrual health and hygiene among Indigenous Australian girls and women: barriers and opportunities. *BMC Womens Health* 2019; **19**(1): 146.	E. Krusz	Health inequities inhibit global development and achievement of the Sustainable Development Goals. One gendered health area, Menstrual Health & Hygiene (MHH), has received increasing attention in Low- and Middle-Income Countries as a barrier to health, wellbeing, and gender equity. Recent anecdotal evidence in Australia highlights that MHH also present challenges to High Income Countries, particularly among underrepresented populations, such as Indigenous Australian peoples, people from low socio-economic backgrounds, or communities that are remotely located. In this article, we chart the emergence of attention to MHH in the Australian context and highlight key considerations for the conduct of research with Aboriginal and Torres Strait Islander Peoples within the culturally- and gender-sensitive area of MHH. Further we draw on insights offered by a partnership between female Aboriginal and Torres Strait Islander leaders, NGO stakeholders, and non-Indigenous researchers. Through a convening (yarning circle) held in March 2018, the group identified multiple socioecological considerations for MHH research and practice, including affordability and access to menstrual products, barriers to knowledge and culturally sensitive education, infrastructure and supply chain challenges, and the necessity of Indigenous-led research and community-driven data collection methods in addressing the sensitive topic. We draw together these insights to develop recommendations for future research, advocacy, and action in Australia.	Menstrual hygiene, Menstrual health, Participatory action research, Australia, Commentary, Indigenous Australians, Aboriginal and Torres Strait Islander Peoples	BMC Womens Health 2019 Vol. 19 Issue 1 Pages 146	2019
Lansbury (2021)		Lived experiences of menstrual health for Indigenous girls in a remote Australian town	N. Lansbury and M. King	Lansbury N, King M. Lived experiences of menstrual health for Indigenous girls in a remote Australian town. *Health Care Women Int* 2021: 1-18.	N. Lansbury	Remote-living and Indigenous Australians can experience menstrual health and hygiene challenges. the topic is not often openly discussed as it is considered private 'women's businesses. We were invited into a remote indigenous town and community to discuss the lived experience of menstruation with female students as well as clinical, educational and community workers. Our yarning circles and interviews collated 16 barriers in four clusters: structural living situation; knowledge, culture and behaviour; discomfort and public life; and finances. targeted efforts by relevant organizations, including schools and government agencies, were identified by participants to better support menstrual health and hygiene without associated pain, ranging moods and a sense of shame.		Health Care Women Int 2021 Pages 1-18	2021 - Accepted
Armour (2020)		The Prevalence and Educational Impact of Pelvic and Menstrual Pain in Australia: A National Online Survey of 4202 Young Women Aged 13-25 Years	M. Armour, T. Ferfolja, C. Curry, M. S. Hyman, K. Parry, K. J. Chalmers, et al.	Armour M, Ferfolja T, Curry C, et al. The Prevalence and Educational Impact of Pelvic and Menstrual Pain in Australia: A National Online Survey of 4202 Young Women Aged 13-25 Years. *J Pediatr Adolesc Gynecol* 2020; **33**(5): 511-8.	M. Armour	**Study Objective:** To explore the prevalence and impact of dysmenorrhea, pelvic pain and menstrual symptoms on young women at school or in tertiary education. **Design and Setting:** Cross-sectional online survey in Australia. **Participants:** A total of 4202 adolescent and young women (13-25 years of age; median age 17 years), having reached menarche, living in Australia, and currently attending school (n = 2421) or tertiary education (n = 1781). **Interventions:** Online survey hosted by Qualtrics between November 2017 to January 2018. Data were collected on sociodemographic data, menstrual cycle characteristics, dysmenorrhea, pelvic pain, and educational and social impact. **Main Outcome Measures:** Information on menstrual and pelvic pain impact, academic absenteeism and presenteeism, impact on non-academic activities and interactions with teaching staff. **Results and Conclusions:** Dysmenorrhea was reported by 92% of respondents. Dysmenorrhea was moderate (median 6.0 on a 0-10 numeric rating scale) and pain severity stayed relatively constant with age [r(s) (3804) = 0.012, P = .477]. Noncyclical pelvic pain at least once a month was reported by 55%. Both absenteeism and presenteeism related to menstruation were common. Just under half of women reported missing at least one class/lecture in the previous three menstrual cycles. The	Dysmenorrhea, Absenteeism, Presenteeism, Education, Pelvic Pain	J Pediatr Adolesc Gynecol 2020 Vol. 33 Issue 5 Pages 511-518	2020

						majority of young women at school (77%) and in tertiary education (70%) reported problems with classroom concentration during menstruation. Higher menstrual pain scores were strongly correlated with increased absenteeism and reduced classroom performance at both school and in tertiary education. Despite the negative impact on academic performance the majority of young women at school (60%) or tertiary education (83%) would not speak to teaching staff about menstruation.			
Armour (2021)		Menstrual Health Literacy and Management Strategies in Young Women in Australia: A National Online Survey of Young Women Aged 13-25 Years	M. Armour, M. S. Hyman, M. Al-Dabbas, K. Parry, T. Ferfolja, C. Curry, et al	Armour M, Hyman MS, Al-Dabbas M, et al. Menstrual Health Literacy and Management Strategies in Young Women in Australia: A National Online Survey of Young Women Aged 13-25 Years. *J Pediatr Adolesc Gynecol* 2021; **34**(2): 135-43.		**Study Objective:** To explore key aspects of menstrual health literacy and menstrual management in young women at school or in tertiary education. **Design:** Cross-sectional online survey. **Setting:** Australia-wide. **Participants:** A total of 4202 adolescent and young women (13-25 years of age; median age 17 years), having reached menarche, living in Australia, and currently attending school (n 5 2421) or tertiary education (n 5 1781). **Interventions:** Online survey hosted by Qualtrics between November 2017 and January 2018. Data were collected on contraceptive use, management strategies, sources of information, and knowledge of menstruation. **Main Outcome Measures:** Information on prevalence and effectiveness of different management strategies, health-seeking behaviour, knowledge about menstruation, and common menstrual disorders such as endometriosis. **Results:** The majority of young women did not seek medical advice for their menstrual symptoms but used information from the Internet (50%) and engaged in self-management, most commonly with over-the-counter medications such as paracetamol (51%) or ibuprofen (52%). Oral contraceptive use was relatively common (35%), and mostly for reduction of menstrual pain (58%). Despite having significant dysmenorrhea, approximately one-half of the participants (51%) thought that their period was normal. Women with higher pain scores were more likely to rate their period as "abnormal" (P ! .0001) but not more likely to consult a doctor (P 5 .13). Only 53% of those at school had heard of endometriosis. **Conclusion:** Self-management of menstrual symptoms is common, but a significant minority of women are underdosing or choosing ineffective methods. Most women do not seek medical advice even when symptoms are severe and cannot identify symptoms suggestive of secondary dysmenorrhea. Improved education on menstruation is vital.	dysmenorrhea, Menstruation, Health literacy, Education, Endometriosis, Pelvic pain	J Pediatr Adolesc Gynecol 2021 Vol. 34 Issue 2 Pages 135-143	2021
Tewhaiti-Smith (2022)		An Aotearoa New Zealand survey of the impact and diagnostic delay for endometriosis and chronic pelvic pain	J. Tewhaiti-Smith, A. Semprini, D. Bush, A. Anderson, A. Eathorne, N. Johnson, et al.	Tewhaiti-Smith J, Semprini A, Bush D, et al. An Aotearoa New Zealand survey of the impact and diagnostic delay for endometriosis and chronic pelvic pain. *Sci Rep* 2022; **12**(1): 4425.	J. Tewhaiti-Smith	Chronic pelvic pain (CPP) causes important negative effects on quality of life. Endometriosis is the most common cause of CPP in females, and diagnostic delay is over six years internationally. Data remain scarce for CPP impact or diagnostic delay in Aotearoa New Zealand. This study used an online survey to explore the impact of CPP on various life domains for those aged over 18. Additionally, for those with an endometriosis diagnosis, diagnostic delay and factors affecting this over time were explored. There were 800 respondent (620 with self-reported endometriosis). CPP symptoms, irrespective of final diagnosis, started prior to age 20 and negatively impacted multiple life domains including employment, education, and relationships. Mean diagnostic delay for those with endometriosis was 8.7 years, including 2.9 years between symptom onset and first presentation and 5.8 years between first presentation and diagnosis. Five doctors on average were seen prior to diagnosis. However, there was a reduction in the interval between first presentation and diagnosis over time, from 8.4 years for those presenting before 2005, to two years for those presenting after 2012. While diagnostic delay is decreasing, CPP, irrespective of aetiology, continues to have a significant negative impact on the lives of those affected.		Sci Rep	2022

Table S1. continued.

Study Characteristics				Study Participants					
**Study Design**	**Methodology**	**Aim**	**Setting of Data Collection**	**Age Range**	**Race/Ethnicity**	**Socioeconomic status**	**Region**	**Urbanity (ie, rural or urban)**	**Sample Size**
Co-designed Qualitative research	Qualitative - Co-design Indigenous led Yarning Circles - Semi-structured interviews	To recommend research priorities, methods, advocacy and action aiming to identify and address the barriers Indigenous Australian women and girls may face when menstruating.	Community setting	Yarning Circle Indigenous participants were primarily from a mature age demographic.	Australian First Nations People	Low socio-economic	Yarning circle convened in Brisbane Queensland	Urban, rural, and remote	12 Aboriginal and Torres Strait Islander women and 8 non-indigenous co-researchers and practitioners.
Co-designed Qualitative research.	Qualitative - Co-design: community lead Yarning circles and interviews.	Are there barriers to menstrual health and hygiene management? If so, what are the girls' proposals to overcome or minimise these barriers?	Community setting	Assigned female at birth age range 10 - 18 years. Adults who worked supporting students.	Indigenous and non-Indigenous girls as well as their support networks who support them to manage their menstruation effectively.	Low socio-economic	Weipa, Far North Queensland	Very remote	Yarning circles with 72 female students plus 15 adult interviews; 7 Indigenous people and 8 non-Indigenous people.
Quantitative Research	Quantitative - Cross sectional online survey	To explore the prevalence and impact of dysmenorrhea, pelvic pain and menstrual symptoms on young women at school or in tertiary education.	Online recruitment	13-25 Years - median range 17 years.	Australian People - 3.2% identify as Aboriginal and Torres Strait Islander	37% lived in low socioeconomic area and 16% lived in a high socioeconomic area.	Australia	Australia wide	4202
Quantitative Research	Quantitative - Cross sectional online survey.	To gain a better understanding of the possible relationship between pain and absenteeism or presenteeism.	Online recruitment	13-25 Years - median range 17 years.	Australian People - 3.2% identify as Aboriginal and Torres Strait Islander	37% lived in low socioeconomic area and 16% lived in a high socioeconomic area.	Australia	Australia wide	4202
Quantitative Research	Quantitative - Cross sectional online survey.	The impact and diagnostic delay of endometriosis and chronic pelvic pain in Aotearoa New Zealand	Online recruitment	18 and over – standard median range of 31.8 years	Cross ethnicity endometriosis majority European (82.7%) and Māori (12.1%). Cross ethnicity CPP majority European (78.3%) and Maori (16.1%).	Most reported university level education and most respondents were in the $501 - $1500 (NZD) per week earnings.	Aotearoa New Zealand	Aotearoa New Zealand wide	800

Table S1. continued.

Menstrual topic area			
**1. Knowledge**	**2. Attitudes/ perception**	**3. Access and barriers**	**4. Personal and Social Impact**
Lack of contemporary, comprehensive puberty education that includes information on menstruation and menstrual hygiene.	Menstruation is considered private ‘women’s business’ in many Aboriginal and Torres Strait Islander cultures, making it a particularly sensitive topic to discuss. Stigma, secrecy and shame involved with discussing menstruation and bleeding may prevent older women from exploring those issues with young people.	Barriers: storing and transporting products, overcrowded housing, multiple residences, lack of privacy for safe keeping of personal items. Financial barriers.	Impact on girls’ self-esteem, sexual and reproductive health, school attendance. Need to improve MHH without distraction from engaging in full societal participation.
Lack of knowledge, puberty-related changes, product preferences, hormonal contraceptives and cultural shame associated with menstruation.	Shame is often an easily felt by Indigenous women when discussing menstruation. Stigma, secrecy and shame involved with discussing menstruation and bleeding.	Barriers to accessing and disposing of menstrual products.	Educational impact was potentially significant
Limited knowledge of menstruation.	Affected by both presenteeism and absenteeism with a detrimental impact on education. Menstrual stigma and a variety of ethical and cultural taboo challenges.	Access to menstrual education is available but effectiveness has not been assessed.	Detrimental impact on education with both absenteeism and presenteeism, concentration problems. Higher menstrual pain scores correlated to higher impact.
Limited knowledge of menstruation and health literacy. Knowledge gained from the internet.	Participants reported problems with classroom concentration during menstruation. Menstrual stigma and a variety of ethical and cultural taboo challenges.	Lack of basic knowledge of menstruation and low health literacy leading to associated overall poorer health outcomes	Impact is most women self-managing their symptoms with the use of ‘over the counter’ pain medications, hormonal contraceptives
Limited and often delayed diagnosis	Endometriosis and CPP a negative impact to work, daily activities and quality of life. Limited awareness	CPP often delayed diagnosis and increased economic burden	Impact of diagnostic delay - significant effects on all social domains, education, work and sexual and other personal relationships.
